# Enhancing Permanency in Children and Families (EPIC): a child welfare intervention for parental substance abuse

**DOI:** 10.1186/s12889-021-10668-1

**Published:** 2021-04-23

**Authors:** Bridget Freisthler, Kathryn Maguire-Jack, Susan Yoon, Elinam Dellor, Jennifer Price Wolf

**Affiliations:** 1 College of Social Work, The Ohio State University, 208 Stillman Hall, 1947 College Road, Columbus, OH 43210 USA; 2 School of Social Work, University of Michigan, Ann Arbor, MI USA; 3 Division of Social Work, Sacramento State University, Sacramento, California USA; 4 Prevention Research Center, Pacific Institute for Research and Evaluation, Berkeley, California USA

**Keywords:** Substance abuse, Substance use disorders, Child maltreatment, Child welfare, Child well-being

## Abstract

**Background:**

Across Ohio, parental substance abuse has contributed to a marked increase in the number of children in foster care. Children exposed to parental substance use have a higher likelihood of physical abuse and neglect, and consequently a variety of physical, psychological and cognitive problems. The Enhancing Permanency in Children and Families (EPIC) program is a collaborative effort between the Ohio State University College of Social Work, two county offices of the Ohio Department of Job and Family Services, two juvenile courts and local behavioral health agencies. The goal of EPIC is to use three evidence-based and evidence-informed practices to reduce abusive and neglectful parenting, reduce addiction severity in parents, and improve permanency outcomes for families involved with the child welfare system due to substance abuse.

**Methods:**

EPIC is a quasi-experimental study. Under the program**,** child welfare-involved adults who screen positive for substances are matched with a peer recovery supporter. Participants are also incentivized to participate in family treatment drug court, medications for opioid use disorders and home-based parenting supports. Participating adults (*N* = 250) are matched with comparison groups from counties participating in a separate intervention (Ohio START) and to those receiving treatment as usual, resulting in a final sample of 750 adults. Primary outcomes including addiction severity, child trauma symptoms, resilience, and attachment are assessed at baseline and at program completion. Additional outcomes include timely access to treatment services, length of placement in out-of-home care and recidivism into the child welfare system.

**Discussion:**

This intervention formalizes cross-system collaboration between child welfare, behavioral health and juvenile courts to support families affected by addiction. The use of three evidence-based or evidence-informed strategies presents the opportunity to determine specific strategies that are most effective for reducing addiction severity. Lastly, the intervention combines several sources of funding to bolster sustainability beyond the life of the Regional Partnership Grant (RPG).

**Trial registration:**

NCT04700696. Registered January 7, 2021-retrospectively registered.

**Supplementary Information:**

The online version contains supplementary material available at 10.1186/s12889-021-10668-1.

## Introduction

## Background

A significant proportion of children in the United States are exposed to substance-using parents, with prior estimates suggesting 12% of children live in a household where at least one parent is abusing or dependent on alcohol or other drugs [1]. Children exposed to parental substance misuse experience higher risks for any array of physical, cognitive, psychological, and social harms [2]. Some of these adverse consequences are likely due to parental drug abuse or dependence being associated with a higher likelihood of child physical abuse or neglect, particularly when the perpetrator is a biological parent [3, 4].

While no conclusive national estimate of the number of children entering the child welfare system due to parental substance use exists, an estimated 1 in 5 children live in a home with an adult who misuses drugs and alcohol [5]. Caseworkers estimate that 50–80% of parents on their caseloads have problems with substance use and that their children are more likely to enter foster care [6–8]. The Fourth National Incidence Study of Child Abuse and Neglect found that drug use was a factor in 9.5% of cases of abuse and 12.5% of all neglect cases [4]. Parents with a diagnosed substance use disorder are more likely to be physically abusive, commit child neglect, and have a higher child abuse potential than those without a diagnosed substance use disorder [9, 10]. In fact, children in these families are two times more likely to be at risk for child maltreatment [11].

This evidence is especially relevant in Ohio, which has the highest rates heroin and synthetic opioid-related deaths in the country [12]. Between 2016 and 2017, Ohio saw an 18.4% increase in the rate of drug overdoses due to prescription opioid misuse. In 2017, there were 46. 3 opioid-related deaths per 100,000 people, more than double the national rate of 13. 3 deaths per 100,000 [12]. While the national rate of heroin overdose decreased between 2016 and 2017, at 9. 2 deaths per 100,000 persons, the rate of heroin deaths in Ohio remains significantly higher than the national rate of 5 deaths for every 100,000 Americans [12].

These trends have overwhelmed Ohio’s child welfare system. Fifty percent of children placed in state custody were due to parental substance use, with nearly half (28%) due to opioid use [13]. Consequently, the number of children placed in custody increased by 11% [13].

### Need for services

Fairfield and Pickaway are adjacent counties in central Ohio. As of the 2010 census, Fairfield County had a population of 146,156, largely composed of Whites (90. 2%), with Blacks representing 6.0% of the population. Southwest of Fairfield is Pickaway County, with a much smaller population of 55,698. Whites make up 94.5% of the population followed by Blacks at 3.4%. Both counties have high rates of opioid-addicted families involved with the child welfare system. The rate of investigations for child maltreatment in Fairfield County is above the state average at 48.09 per 1000 children [14]. Though the rate of child maltreatment investigations is lower in Pickaway County (14.29 per 1000), the number of children in out-of-home care rose over 200% from 23 in 2013 to 73 in 2018 [14, 15]. According to a survey conducted by the Public Children Services Association of Ohio (PCSAO), 79% of families involved in Fairfield County Children’s Services and 67% of families in Pickaway County Children Services have identified substance use problems, both higher than the state-wide average of 50% [16]. In Pickaway County, all the substance-involved families were due to opioid (including heroin) misuse, while 58% of all cases in Fairfield County involved opioids [16]. These rates are over twice the state average of 28% [16]. Thus, while opioid addiction is a particular concern, all types of substance use are affecting children involved in the child welfare system.

The two county agencies have seen a significant increase in the number of children on their caseloads. This increase is attributed to the increase in parental substance abuse and has contributed to a higher number of children in foster care and rising foster care costs. In 2013, Pickaway County Children Services placed 10 children in foster care in contrast, a total of 46 children were placed in foster care in 2018 [16]. In Fairfield County, the overall number of children in agency custody has remained steady from previous years, yet the costs to house these children has increased dramatically due to the needs of the children. In 2014, the agency spent $1.6 million on out-of-home placement costs, by 2018, the cost to house the same average number of children jumped to nearly $ 3.1 million [14]. Many of these children are placed with relatives who may be ill-equipped to address the specific needs of children exposed to substance use problems. As there are limited non-relative foster homes available, children are often placed several hours away from the agency creating challenges for staff to meet face-to-face requirements and provide regular visitation between parents and children.

### Rationale for study

Both counties have taken significant steps to address the needs of families, including the implementation of Ohio START (Sobriety, Treatment, and Reducing Trauma) [17].

Fairfield and Pickaway counties also have Family Treatment Drug Courts (FTDC) in operation. FTDC includes frequent court hearings, judicial monitoring, substance abuse treatment, drug testing, and rewards and sanctions linked to parental compliance with service plans [18]. However, only six residential or intensive outpatient substance use providers are located within the boundaries of the two counties, with only one providing residential or detoxification facilities. While a 31bed facility recently opened in Pickaway county [19], the demand for detoxification beds still outweighs the current supply. Fairfield County has little infrastructure to support the use of medications for opioid use disorders (MOUD) and has not used it systematically for child welfare families. Further, while service providers and local governments tend to think in terms of county lines, families do not use that same thought process when seeking help. In this way, the patchwork structure of alcohol and other drug (AOD) services inhibits the ability of both counties to address the AOD needs of families involved in the child welfare system.

Funded by the federal Regional Partnership Grant (RPG) Program, the Enhancing Permanency in Children and Families (EPIC) is a partnership between child welfare, juvenile count court  and behavioral health, to holistically address substance misuse and associated parenting needs of child welfare-involved families.

## Methods / design

The overall goals and objectives of the intervention are:

1. Increase timely access to services among substances abusing parents involved in the child welfare system in Fairfield and Pickaway counties
  1.1 Develop procedures to coordinate services between the child welfare system, substance abuse and behavioral health providers, and juvenile/family court in a specified single MOU.
  1. 2 Conduct substance abuse and trauma-exposure screening and assessment within 30 days of entering the child welfare system.
  1. 3 Reduce the wait time between referral to services and initiation of substance use and behavioral health services.
2. Enhance child safety and improve permanency
  2.1 Reduce length of stay in out-of-home placement for children in EPIC program compared to substance-affected families not receiving EPIC.
  2. 2 Increase rates of reunification among families involved in EPIC compared to substance-affected families not receiving EPIC.
  2. 3 Reduce recidivism for child welfare investigations and re-entries into foster care among parents receiving EPIC compared to substance-affected families not receiving EPIC.
  2.4 Identify which intervention components implemented via EPIC were more likely to increase positive outcomes for families.
3. Increase child, parent, and caregiver well-being
  3.1 Decrease substance use/abuse addiction severity among parents and the percentage of parents completing substance abuse treatment who maintained abstinence post-treatment.
  3. 2 Reduce trauma symptoms experienced by children.
  3. 3 Increase resilience and attachment in children.
  3.4 Increase knowledge about trauma-exposure among kinship caregivers.
  3.5 Improve parenting among kinship caregivers.

### Study components

EPIC participants are incentivized to participate in (1) family treatment drug court (FTDC), with medications for opioid use disorders (MOUD); (2) peer recovery support and (3) home-based parenting supports based on the Nurturing Parenting Program [20].

#### FTDC with MOUD

FTDC is considered the most effective intervention to improve substance abuse treatment initiation and completion in child welfare populations [21]. Parents who participate in FTDC are more likely to enter substance use treatment, get into treatment quicker, stay in treatment longer, and complete treatment more often [22–24]. Additionally, families who complete FTDC are more likely to be reunified with their children as compared to parents who do not participate [22–25]. To bolster the effects of FTDC, EPIC participants also have access to MOUD, which has been shown to reduce withdrawal symptoms, lower the risk of relapse, address cravings, and provide medical supervision for individuals struggling with addiction [26]. Further, MOUD is associated with an increased likelihood that children will remain in their parents’ custody [27].

*Peer recovery supporters*—also called “recovery coaches”— are advocates for parents and their primary focus is to help get parents into treatment and support them so that they stay there. Peer recovery supporters are certified by the Ohio Department of Mental Health and Addiction Services [28] and must complete 40 h of in-person training, 16 h of online training, and pass a test. They are individuals who formerly had substance use issues, but have been clean and sober for at least 3 years. Peer recovery supporters provide comprehensive clinical assessments, service planning, referral, advocacy, case management, and outreach services. Peer recovery supporters have had great success in reducing substance use and improving child welfare outcomes. Peer recovery supporters improve access to substance use treatment, decrease time children spend in out-of-home care, increase the odds of reunification, decrease maltreatment recidivism, and reduce racial disparities in reunification [29–31]. Under EPIC, peer recovery supporters are trained and supervised by two partner behavioral health agencies, Ohio Guidestone and Integrated Services. Peer recovery supporters make individual or joint home visits with child welfare caseworkers and are otherwise available via phone and text as needed. They also attend multi-disciplinary child welfare team meetings to provide expert consultation on cases.

The third and final component of EPIC is *parenting support (Relational Skill Building)* based on the Nurturing Parenting Program (NPP) [20]. NPP focuses on parental expectations of children, empathy toward children’s needs, alternatives to corporal punishment, appropriate parent-child roles, and children’s power and independence [32]. Under EPIC, the program is further enhanced with modules specific to substance use. For example, unexpected challenges related to administering naloxone, a drug that is used to revive individuals who have overdosed on opioids and for kinship caregivers, ways to maintain safe relationships with biological parents. Under EPIC, parents and kinship caregivers may participate in the home-based relational skill building intervention.

### Participant recruitment, eligibility and group assignment

**Fig. 1:**
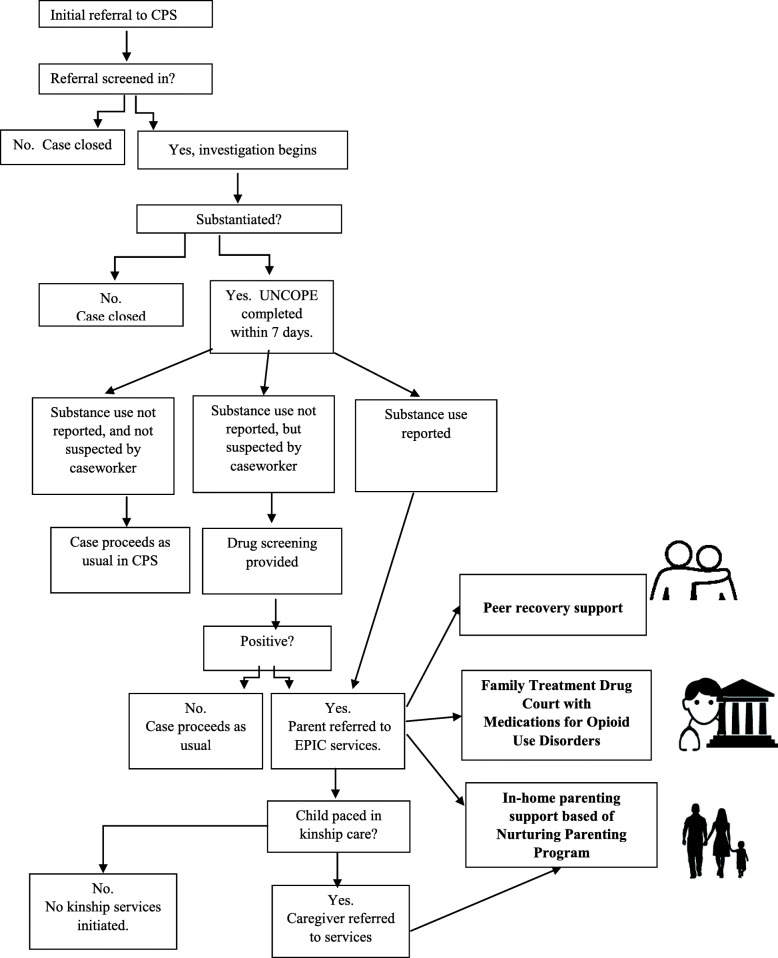
Enhancing Permanency in Children and Families (EPIC): Case Flow. Parents complete the UNCOPE assessment within 7 days of substantiated report of child abuse or neglect. Parents receive a drug screen if they self-identify on the UNCOPE or a caseworker suspects substance use. Parents who screen positive are eligible for EPIC and are 1) linked to a peer recovery supporter, 2) referred to family treatment drug court, with the option of medications for opioid use disorders, and 3) receive in-home parenting based on the Nurturing Parenting Program. Participants work with assigned caseworkers to determine the most appropriate combination of services

Participants are recruited from Fairfield and Pickaway County Children Services. The overall case flow is provided in Fig. 1. Within 7 days of the initial child welfare investigation, parents complete the UNCOPE assessment, a universal screening tool for substance use administered to all parents entering the child welfare system [33]. Parents who score a 3 or higher on the UNCOPE, are considered eligible for EPIC and receive a drug screen. Eligible parents are alternated into EPIC, OhioSTART or treatment as usual (TAU). Ultimately, assignment to EPIC is determined based on caseload, but is typically offered to every third case in Fairfield county and every other case in Pickaway county. Parents who consent to EPIC are connected to peer recovery supporters who mentor parents through the process. Parents are also referred to FTDC with the option to receive MOUD, and lastly when children are placed at home with parents or with kinship caregivers, relational skill building services that include financial assistance for child care, respite and transportation services. EPIC participants receive substance abuse and behavioral health treatment services through local providers including from two partner agencies: Integrated Services for Behavioral Health and Ohio Guidestone.

To incentivize participation in FTDC, parents receive a $200 gift card upon completing their substance abuse assessment and an additional $200 retails gift card for every 3 months they remain involved with the court. Parents may consent to one or all three components of EPIC based on the specific needs of each family, however all parents receive intensive case management services, including frequent home visits from caseworkers and peer recovery supporters. All procedures involving human participants were approved by The Ohio State University’s Institutional Review Board (#2018B0001).

### Consent and withdrawal from study

Child welfare caseworkers obtain written informed consent and release of information from all parents at intake prior to study enrollment. Kinship caregivers consent for their own participation. Participation is voluntary and participants are free to withdraw from the study at any time without consequences to their child welfare cases.

### Collaboration, coordination and cross-training

Child welfare-involved parents with substance use disorders must navigate multiple systems including behavioral health, and juvenile/family courts in order to complete case plans. To facilitate collaboration and coordination of services for EPIC, all partner agencies entered into one comprehensive memorandum of understanding (MOU) to jointly administer and implement EPIC. All partner agencies further agreed to use the Needs Portal [34], a hybrid web-based resource and referral and Management Information System (MIS), as the organizing body for communication around each case. The Needs Portal is used to create referrals for services, track dates of service provision, and record responses to the UNCOPE and trauma screening and assessment tools. The system works by opening a ‘ticket’ for each case which allows caseworkers to complete substance abuse and trauma assessments and indicate essential information about service requests. Service providers respond to requests and indicate when services have been initiated and peer recovery supporters are able to log visits with participants, all within the same environment. Caseworkers, service providers, and the evaluation team can see this information. Lastly, the Needs Portal provides differential access to the system based on the role of the provider to limit access of information based on a person’s assigned role in the case (i.e., caseworker, therapist, peer recovery supporter).

Prior to implementation, partner agencies also received cross-system training such that child welfare workers become more knowledgeable of substance misuse and trauma-informed care, peer recovery supporters understand the child welfare system and processes, and family drug court coordinators and MOUD providers have a thorough understanding of the child welfare system. Representatives from each system are also members of a Steering Committee that meets every other week, to resolve any implementation related problems and address barriers.

### Evaluation design

To evaluate EPIC, a quasi-experimental design will be employed through a two-stage sampling procedure. This design provides the ability to assess (1) the effects of EPIC on access to services for the families in the two intervention counties, and (2) the independent effects of additional services provided under EPIC that may be over and above interventions provided by Ohio START and treatment as usual (TAU). In the first stage, two comparison counties will be identified for each of the two intervention counties. One comparison county will be part of the Ohio START program while the second will be a county that has no major interventions to address substance use among child welfare families. Counties are matched based on child population size, rate of child protective services referrals, percent of naloxone administrations per adult population, percent white, percent poverty, child welfare tax levy, and to the extent possible, behavioral health service availability. During the second stage, EPIC families (*N* = 250) are matched with substance using families in Ohio START (*N =* 250) and those receiving treatment as usual (*N =* 250).

### Outcome measures

All outcome measures are assessed at baseline and again at 6 months.

#### Sample characteristics

To describe the sociodemographic characteristics of participants, all surveys include demographic information (e.g., biological sex, age, race/ethnicity, nativity). In addition, a clinical description of the sample will be provided using the 10-item Center for Epidemiological Studies-Depression (CES-D) scale for depressive symptoms [35], the Perceived Stress Scale (PSS) [36] and perceived availability of social support using the Interpersonal Support Evaluation List (ISEL) [37]. *Timely access to services* is measured using dates of substance abuse screenings, trauma assessments and treatment service provision recorded in the Needs Portal.

*Child safety and permanency* outcomes are operationalized as (1) the number of days in out-of-home care, (2) whether the family re-unifies in out of home care and (3) the number of new child welfare referrals occurring after the initial referral that led to program participation using administrative child welfare data from the statewide automated child welfare information system (SACWIS).

#### Parent and child well-being outcomes

Addiction severity is measured using the drug and alcohol use items in the Addiction Severity Index-Self Report (ASI-SR) [38]. Trauma symptoms in children are measured using the Children’s Trauma Assessment Center (CTAC) trauma screening checklist [39]. The CTAC—separately for children ages 0–5 and 6–18)—can be administered by clinicians and child welfare caseworkers in evaluating children for exposure to trauma and trauma-related symptoms. The effect of sensory processing on functional performance among children 0–6 months and 7–17 months is measured using version 1 and 2 respectively of the Infant Toddler Sensory Profile (ITSP) [40], behavioral and emotional problems in younger children (18 months to 5 years) and in school age children and adolescents (6 to 18 years) are measured using the Child Behavior Checklist (CBCL) [41] and parenting skills is measured using the 40-item Adult Adolescent Parenting Inventory, version 2 (AAPI-2) [42]. The AAPI-2 breaks down into five parenting behavior subscales: expectations of children, parental empathy towards children’s needs, use of corporal punishment, parent-child family roles, and children’s power and independence. Lastly, resiliency and attachment are measured using the Protective Factors Survey [43].

### Data sources and data collection procedures

Data are available from three different sources: 1) the Needs Portal, 2) SACWIS archival data, and 3) EPIC survey data. Needs Portal data are used to track fidelity indicators in real benchmark dates over the course of implementation. SACWIS data are obtained quarterly from Ohio Department of Job and Family Services (ODJFS) via an encrypted password-protected USB drive. This is administrative data that are routinely collected as part of the investigative and intervention process resulting from a referral for a child abuse and neglect investigation. The final source of data are EPIC family surveys administered at the time of enrollment and again at 6 to 12 months following program completion. Parents complete the survey for themselves and for one focal child. Respondents receive a $25 gift card for completing each survey.

### Statistical plan

Baseline descriptive statistics including sociodemographic characteristics, addiction severity and child welfare allegations will be provided for EPIC participants and comparison groups. For continues variables, means and SDs will be provided and for categorical variables, counts and percentages are provided.

Due to the likelihood of censored measures when families are still involved in the child welfare system, survival analysis will be utilized to assess program effects on length of stay in out of home care. Logistic regressions will be used to estimate the relationship between activities and family was reunification, subsequent child maltreatment investigations, and subsequent child maltreatment substantiations. A series of linear regressions will be used to estimate the relationship between our outcome measures and various independent variables (e.g., age, biological sex). Additionally, a paired samples t-test will be conducted to examine the changes in mean scores in child, parent and caregiver well-being outcomes (e.g. ASI or CBCL) between baseline and 6-months follow-up.

#### Power analysis

We performed a series of power analyses using G*Power [44] and for each analytic method. Assuming a small effect size of 0. 2, we will be able to reject the null hypothesis with probability (power) of 1.00 (survival analysis, *N* = 750), 0.81 (logistic regression, *N* = 750), 0.88 (paird t-tests; *N* = 250) and 0.99 (linear regression, *N* = 250). The Type I error probability associated with these tests is 0.05. A detailed report is presented in Additional file 1.

### Patient and public involvement

The design of this intervention did not involve patients, however, the public’s experiences navigating the child welfare and behavioral health systems was central to formulating research questions, outcome measures as well as the choice of evidence-based or evidence informed practices selected. The activities conducted as part of EPIC is of interest to partners and other public child welfare agencies in Ohio and across the United States. To that end we maintain a dissemination website (https://u.osu.edu/epic/) that acts as a central repository of information and resources.

## Discussion

The EPIC program addresses current deficiencies in substance abuse treatment services for child welfare involved parents by creating an inter-agency partnership with cross-sectoral linkages to address the unique needs of these families. Child welfare agencies, local behavioral health agencies and the courts work jointly to administer and implement EPIC, using the Needs Portal, a management information system for communication and coordination around each case. EPIC holistically addresses substance abuse and associated parenting needs through evidence-based or evidence informed interventions, including FTDC and MOUD, peer recovery support, and parenting supports based on the Nurturing Parenting Program.

EPIC is intended to be a long-term and critical component in the fight against the opioid epidemic in Ohio. We expect to learn the most effective strategies for increasing timely access to services, enhancing child safety and permanency and increasing parent, child and caregiver wellbeing. In this way, the partnership is expected to help build infrastructure and a system of care for substance abusing parents in the child welfare system. By fostering collaborations with County children services, behavioral health experts and County juvenile courts we hope to enhance the sustainability of EPIC. These efforts are further bolstered by a braided funding approach that uses RPG grant funds, Medicaid eligible services and other state and local funding sources. On the individual level, parents are expected to receive timely access to services, reduce substance abuse addiction and reduce recidivism for child welfare investigations and improve resilience and attachment among participating families.

## Supplementary Information

**Additional file 1.**

## Data Availability

Data generated from this intervention will be available following study completion from the corresponding author on reasonable request.

## Abbreviations

AAPI-2: Adult Adolescent Parenting Inventory, version 2
ASI-R: Addiction Severity Index-Self Report
AOD: Alcohol and other drugs
CES-D: Center for Epidemiological Studies-Depression scale
CBCL: Child Behavior Checklist
CTAC: Children’s Trauma Assessment Center trauma screening checklist
EPIC: Enhancing Permanency and Children and Families
FTDC: Family Treatment Drug Court
ITSP: Infant Toddler Sensory Profile
ISEL: Interpersonal Support Evaluation List
MIS: Management Information System
MOUD: Medications for opioid use disorders
NPP: Nurturing Parenting Program
ODJFS: Ohio Department of Job and Family Services
Ohio START: Ohio Sobriety, Treatment, and Reducing Trauma
PCSAO: Public Children Services Association of Ohio
PSS: Perceived Stress Scale
RPG: Regional Partnership Grant
SACWIS: Statewide Automated Child Welfare Information System
TAU: Treatment as usual
