# Supplementary material for: Enhancing Permanency in Children and Families (EPIC): a child welfare intervention for parental substance abuse
Source: BMC Public Health. 2021 Apr 23;21:780. doi: 10.1186/s12889-021-10668-1 (PMC8063333; doi:10.1186/s12889-021-10668-1)
Supplement: Supplementary file 1 — Additional file 1. [file 12889_2021_10668_MOESM1_ESM.docx]

**Enhancing Permanency in Children in Families: Power Analysis**

**Goal 1** : Increase timely access to services among substances abusing parents involved in the child welfare system in Fairfield and Pickaway counties

1.1 Develop procedures to coordinate services between the child welfare system, substance abuse and behavioral health providers, and juvenile/family court in a specified single MOU.

1.2 Conduct substance abuse and trauma-exposure screening and assessment within 30 days of entering the child welfare system.

1.3 Reduce the wait time between referral to services and initiation of substance use and behavioral health services.

**Analysis plan**: Qualitative Comparative Analysis (QCA).

*This approach was designed for midsized N (10 to 50 cases). While we do not perform power analysis per se for this qualitative approach, we will follow prior convention and apply various robustness tests for our N QCA, including changing consistency and frequency thresholds, and randomly deleting cases (*[*Emmenegger, Schraff, and Walter 2014*](https://journals.sagepub.com/doi/full/10.1177/0049124120914955)*;*[*Maggetti and Levi-Faur 2013*](https://journals.sagepub.com/doi/full/10.1177/0049124120914955)*). We will include a robustness table (*[*http://smr.sagepub.com/supplemental/)*](http://smr.sagepub.com/supplemental/) *in the results section of all publications which offers an overview of the robustness of findings.*

**Goal 2. Enhance child safety and improve permanency**

2.1 Reduce length of stay in out-of-home placement for children in EPIC program compared to substance-affected families not receiving EPIC (**survival analysis).**

2.2 Increase rates of reunification among families involved in EPIC compared to substance-affected families not receiving EPIC **(logistic regression).**

2.3 Reduce recidivism for child welfare investigations and re-entries into foster care among parents receiving EPIC compared to substance-affected families not receiving EPIC **(logistic regression).**

2.4 Identify which intervention components implemented via EPIC were more likely to increase positive outcomes for families **(paired t-tests).**


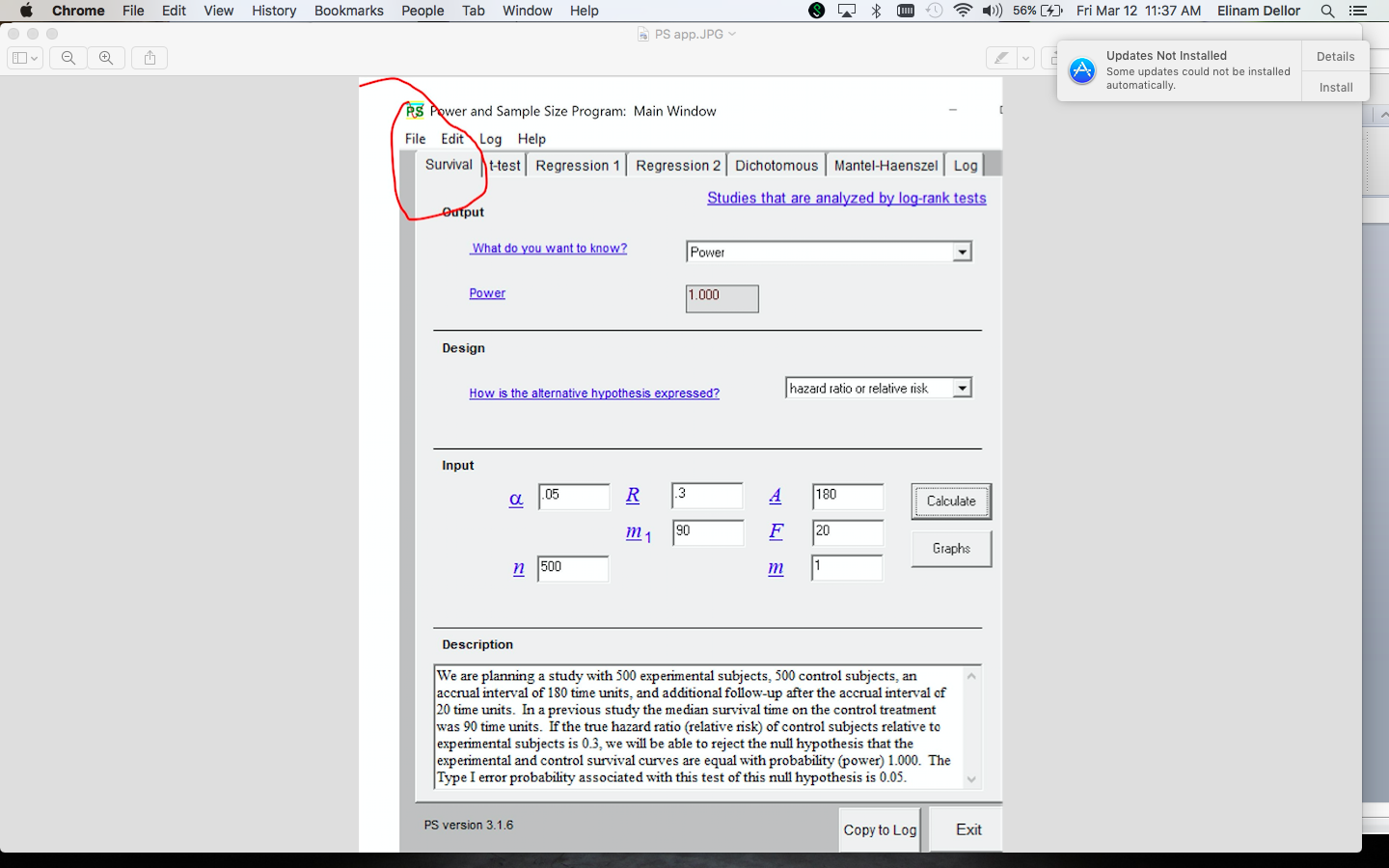
**(2.1) Survival Analysis:**

*We are conducting a study with 750 subjects (250 in the treatment group, 250 the first comparison group [Ohio START] and 250 in the second comparison group [treatment as usual]) in which we will regress length of time in out of home placement against participation in EPIC, Ohio START or treatment as usual. Prior data indicate the standard deviation of program participation is 1 and the standard deviation of the regression errors will be 1. Assuming a small effect size of 0.2, we will be able to reject the null hypothesis that this slope equals 0 with probability (power) of 1.00. The Type I error probability associated with this test of this null hypothesis is 0.05*.

**(2.2, 2.3): Logistic regression**

**Options:** Large sample z-Test, Demidenko (2007) with var corr

**Analysis:** Post hoc: Compute achieved power

**Input:** Tail(s) = Two

Odds ratio = 1.3

Pr(Y=1|X=1) H0 = 0.2

α err prob = 0.05

Total sample size = 750

R² other X = 0

X distribution = Normal

X parm μ = 0

X parm σ = 1

**Output:** Critical z = 1.9599640

Power (1-β err prob) = 0.8155328

*We are conducting an analysis with 750 subjects (250 in the treatment group, 250 the first comparison group [Ohio START] and 250 in the second comparison group [treatment as usual]) in which we will separately regress reunification and recidivism on program type. Assuming a small effect size of 0.2, we will be able to reject the null hypothesis that there is no difference in reunification or recidivism by program type with a probability (power) of 0.82 The Type I error probability associated with this test of this null hypothesis is 0.05.*

**(2.4)T-tests - Means: Difference between two dependent means (matched pairs)**

**Analysis:** Post hoc: Compute achieved power

**Input:** Tail(s) = Two

Effect size dz = 0.2

α err prob = 0.05

Total sample size = 250

**Output:** Noncentrality parameter δ = 3.1622777

Critical t = 1.9695369

Df = 249

Power (1-β err prob) = 0.8829954

*We are conducting an analysis with 250 pairs of subjects. Prior data indicate that the difference in the response of matched pairs is normally distributed with standard deviation 1.00. If the true difference in the mean response of matched pairs is 0.20, we will be able to reject the null hypothesis that this response difference is zero with probability (power) of 0.88. The Type I error probability associated with this test of this null hypothesis is 0.05.*

**Goal 3: Increase child, parent, and caregiver well-being**

3.1 Decrease addiction severity among parents and the percentage of parents completing substance abuse treatment who maintained abstinence post-treatment (**matched t-tests and linear regressions**).

3.2 Reduce trauma symptoms experienced by children **(matched t-tests and linear regressions).**

3.3 Increase resilience and attachment in children **(matched t-tests and linear regressions).**

3.4 Increase knowledge about trauma-exposure among kinship caregivers **(matched t-tests and linear regressions).**

3.5 Improve parenting among kinship caregivers **(matched t-tests and linear regressions).**

**T tests - Means: Difference between two dependent means (matched pairs)**

**Analysis:** Post hoc: Compute achieved power

**Input:** Tail(s) = Two

Effect size dz = 0.2

α err prob = 0.05

Total sample size = 250

**Output:** Noncentrality parameter δ = 3.1622777

Critical t = 1.9695369

Df = 249

Power (1-β err prob) = 0.8829954

We are planning a study with 250 pairs of subjects. Prior data indicate that the difference in the response of matched pairs is normally distributed with standard deviation 1.00. If the true difference in the mean response of matched pairs is 0.20, we will be able to reject the null hypothesis that this response difference is zero with probability (power) of 0.88. The Type I error probability associated with this test of this null hypothesis is 0.05.

**F tests** - Linear multiple regression: Fixed model, R² increase

**Analysis:** Post hoc: Compute achieved power

**Input:** Effect size f² = 0.2

α err prob = 0.05

Total sample size = 250

Number of tested predictors = 2

Total number of predictors = 10

**Output:** Noncentralty parameter λ = 50.0000000

Critical F = 3.0335979

Numerator df = 2

Denominator df = 239

Power (1-β err prob) = 0.9999987

We are conducting a study with 250 subjects, comparing pre and post test data. Assuming a small effect size of 0.2, we will be able to reject the null hypothesis that this response difference is zero with probability (power) of 0.99. The Type I error probability associated with this test of this null hypothesis is 0.05.

***References:***

Emmenegger, P., Schraff, D., Walter, A.. 2014. “QCA, the Truth Table Analysis and Large-N Survey Data: The Benefits of Calibration and the Importance of Robustness Tests.” Compasss Working Paper 2014-79

Maggetti, M., Levi-Faur, D.. 2013. “Dealing with Errors in QCA.” Political Research Quarterly 66:198–204.
